# Comparison of Strength Training Interventions on Functional Performance in Frail Nursing Home Residents

**DOI:** 10.3390/healthcare14030303

**Published:** 2026-01-26

**Authors:** Helena Vila, Carmen Ferragut, Luis Javier Chirosa, Virginia Serrano-Gómez, Óscar García-García, Daniel Jerez-Mayorga, Ángela Rodríguez-Perea, José María Cancela

**Affiliations:** 1 Department of Specific Didactics, Faculty of Education and Sport Sciences, University of Vigo, 36005 Pontevedra, Spain; evila@uvigo.es (H.V.); oscargarcia@uvigo.es (Ó.G.-G.);; 2 Area of Physical and Sports Education, Faculty of Medicine and Health Sciences, Department of Biomedical Sciences, University of Alcalá, 28801 Madrid, Spain; 3 Department of Physical Education and Sports, Faculty of Sport Sciences, Sport and Health University Research Institute (iMUDS), University of Granada, 18071 Granada, Spain; lchirosa@ugr.es (L.J.C.); djerezmayorga@ugr.es (D.J.-M.); arodrp@unileon.es (Á.R.-P.); 4 Exercise and Rehabilitation Sciences Institute, Faculty of Rehabilitation Sciences, Universidad Andres Bello, Santiago 7591538, Chile; 5 Faculty of Physical Activity and Sports Sciences, Department of Physical Education and Sport, Universidad de León, 24007 León, Spain

**Keywords:** strength training, physical performance, frailty, quality of life, functional electromechanical dynamometer

## Abstract

**Background/Objectives:** Frailty and functional decline represent major challenges for aging populations, particularly among institutionalized older adults. Preserving functional capacity is essential to maintain autonomy, mobility, and quality of life. This study aimed to compare the effects of two strength training interventions—functional electromechanical dynamometer (FEMD) training and weighted vest training—on peak concentric and eccentric force during the sit-to-stand task, as well as on functional performance and body composition in frail nursing home residents. **Methods:** A pilot quasi-experimental study with a non-randomized control group was conducted in 19 older adults (mean age: 86.3 ± 5.8 years). Participants were allocated to FEMD training (EG1, n = 6), weighted vest training (EG2, n = 6), or a control group (CG, n = 7). Training was performed twice weekly for eight weeks. Assessments included body composition, handgrip strength, 30 s chair stand test, 3 m walking speed, and peak concentric and eccentric force during the sit-to-stand movement. Data were analyzed using mixed-model ANOVA and complementary within-group analyses. **Results:** No significant group × moment interactions were observed. However, EG1 demonstrated significant within-group improvements in chair stand performance (+4.8 repetitions, *p* = 0.006), walking speed (+0.1 m·s^−1^, *p* = 0.030), concentric peak force (+46.5%, *p* = 0.008), and eccentric peak force (+34%, *p* = 0.047). EG2 showed a smaller but significant increase in eccentric peak force (+6.1%, *p* = 0.019), without functional improvements. Body composition changes were modest, with EG1 showing increases in weight and BMI without concomitant fat mass gains. **Conclusions:** In this pilot quasi-experimental study, functional electromechanical dynamometer-based training was associated with improvements in neuromuscular performance, particularly concentric peak force. However, no significant group × moment interactions were observed, indicating that differential effects between interventions cannot be established. Functional improvements should be interpreted cautiously. The present results should therefore be considered exploratory and hypothesis-generating. These findings suggest that FEMD-based training may be a feasible and potentially beneficial functional strength training strategy for frail institutionalized older adults, which should be confirmed in adequately powered randomized controlled trials.

## 1. Introduction

As global populations continue to age, the prevalence of frailty and functional decline among older adults has become a significant public health concern [1]. Sedentary life-styles, often intensified by institutional living environments, accelerate the deterioration of physical function, increase the risk of falls, and reduce quality of life [1]. Frailty, characterized by decreased physiological resilience, makes older adults particularly vulnerable to functional loss, disability, and dependency. Consequently, interventions that counteract frailty by improving muscle strength and motor control are essential for sustaining health and delaying functional deterioration.

Walking speed is a critical indicator of health and functionality in older adults, as it is directly associated with morbidity and mortality [2]. Gait speed has been proposed as a reliable proxy for frailty status and a predictor of adverse health outcomes, including hospitalization and mortality [3,4]. Reduced walking speed is linked to higher risk of falls and loss of independence [5], highlighting the importance of maintaining functional capacity to preserve quality of life [6]. Since walking speed depends on muscle strength and endurance, engaging in strength exercises and mobility-enhancing activities can mitigate age-related declines and improve quality of life [7,8].

Strength training has emerged as a fundamental strategy for preventing and counteracting frailty in older adults [9]. Evidence consistently highlights the benefits of resistance training for improving muscle strength, mass, and overall functional capacity [7,10,11]. Furthermore, this type of exercise is recognized as an evidence-based intervention to delay frailty progression, enhance independence, and improve multidimensional health outcomes, including mental well-being and self-perceived quality of life.

Despite these benefits, there remains a need for effective and adaptable exercise protocols that can be safely implemented in institutional settings. Electromechanical and weighted strength training methods offer practical and individualized approaches to enhance physical and functional capacities in frail older populations. By focusing on frail institutionalized adults, this research explores how these training modalities contribute to maintaining independence and overall health status, addressing critical aspects of frailty management and quality of life.

The eccentric phase of muscle contraction, during which muscles lengthen under tension, has garnered attention for its unique advantages [12]. For example, programs combining strength and balance training have been shown to increase walking speed, thereby improving mobility and reducing fall risk in older adults [13].

Traditional methods such as free weights [14] and resistance machines [15] require engagement of both concentric and eccentric phases. The concentric phase occurs when the muscle shortens to produce external work, while the eccentric phase involves muscle lengthening, generating both positive and negative work [16].

New technologies, such as Functional Electromechanical Dynamometry (FEMD), represent advanced methodological approaches for quantifying neuromuscular performance variables—specifically strength, power, and velocity—during functional tasks [17]. Compared to traditional isokinetic or handheld dynamometry, FEMD offers superior ecological validity by replicating real-life or sport-specific motor actions [17,18].

The eccentric phase allows training with heavier loads—20% to 50% greater than the concentric contractions [19]. Strength gains from eccentric training are often superior to conventional methods [20]. Additionally, eccentric exercise improves muscle and joint flexibility by increasing the extensibility of fibers and connective tissue, thereby reducing injury risk, optimizing range of motion, and enhancing walking speed [21,22]. Studies show that eccentric contractions generate greater muscular forces with lower energy costs [23,24], which is particularly advantageous for older adults experiencing declines in muscle mass and metabolic efficiency [24]. Eccentric training also promotes neuromuscular adaptations such as improved intermuscular coordination and greater motor unit activation, both critical for motor control, stability, and fall prevention [25,26]. High-intensity eccentric contractions accelerate strength gains and hypertrophy, directly contributing to improvements in walking speed [24].

Although eccentric training can initially cause acute muscle damage and delayed onset muscle soreness (DOMS), these responses are part of the adaptation process and diminish with progressive training [27]. This phenomenon, known as the “repeated bout effect”, reduces susceptibility to muscle damage over time, allowing long-term benefits to outweigh the initial discomfort [27].

Given this evidence, eccentric training appears to be an effective strategy for optimizing physical performance, improving metabolic and cardiovascular health, and preventing injuries in older adults. Therefore, this study aimed to compare the effects of two strength interventions—functional electromechanical dynamometer training and weighted training—on peak force during concentric and eccentric phases of the sit-to-stand movement in older adults residing in a nursing home. Additionally, we evaluated the impact of both interventions on functional capacity (gait speed, hand-grip strength, and sit-to-stand performance) and body composition. We hypothesized that eccentric-focused strength training would lead to greater improvements in peak force compared to traditional combined concentric-eccentric training.

We hypothesized that strength training performed with a functional electromechanical dynamometry would lead to greater improvements in peak force compared to similar exercises performed with a weighted vest.

## 2. Materials and Methods

### 2.1. Participants

Of the 22 older adults initially recruited, 19 completed the study. Three participants voluntarily withdrew due to reasons such as leaving the residence or experiencing physical discomfort, resulting in a final sample of 19 individuals (age: 86.3 ± 5.8 years; sex (female/male): 12/7). Participants were assigned to one of three groups: Group 1 (EG1, n = 6), which performed strength training using FEMD; Group 2 (EG2, n = 6) which trained with weighted vests; and a Control Group (CG, n = 7), which received no intervention (e.g., “continued with their usual daily activities and standard nursing home care”). Regarding group allocation, a participant-preference quasi-experimental design was used [28]. The control group consisted of individuals who chose not to participate in either of the two training programs. The two experimental groups were assigned by the facility’s physiotherapist based on organizational criteria. Importantly, the research team did not participate in the allocation process at any point. Inclusion criteria included the ability to perform the exercises proposed in the intervention and the ability to attend 85% of the sessions. Exclusion criteria included any condition that contraindicated strength training, difficulty following the instructor’s instructions, or expressing a desire to withdraw from the program. Procedures, potential risks or discomfort associated with the experiments were explained to participants, who then gave their written informed consent. The study design and protocol adhered to the tenets of the Declaration of Helsinki and was approved by the University Review Board (CEIC-14-1009-17). The sample size calculation revealed that 18 participants [29,30] were estimated to be sufficient for this study to detect an effect size of 0.7 (α = 0.05; 1 − β = 0.90) (G*power V3.1, Dusseldorf University, Germany). Based on this estimation, the study was designed with three conditions, allocating six participants to each group (n = 6). This distribution was considered appropriate given the expected large effect size, the use of repeated measures to enhance statistical sensitivity, and practical constraints related to participant recruitment in this specific population. Such an approach ensures methodological rigor while maintaining feasibility in a controlled experimental setting.

### 2.2. Experimental Design and Protocol

A pilot quasi-experimental study with a non-randomized control group was conducted to compare the effects of two strength training interventions (FEMD and weighted vest training) on execution speed and peak force during the concentric and eccentric phases of the sit-to-stand movement.

Baseline and post-intervention assessments were carried out over two no-consecutive days each, due to the number of participants. Height was measured first, followed by body composition. Subsequently, the hand grip strength test was performed, along with the 30 s sit-to-stand test and a 3 m walking speed test. On the first day, participants underwent a familiarization session with the FEMD, performing four sets of six repetitions. On the second day, the sit-to-stand test was performed using the FEMD, with a 48 h rest period between pre- and post-intervention assessments. A 48 h interval was selected to ensure adequate recovery and to minimize potential fatigue or acute learning effects associated with the familiarization protocol. Additionally, prior research using electromechanical dynamometry and sit-to-stand testing in older adults has employed rest periods of 24–48 h between familiarization and baseline testing without adverse effects on test reliability or performance outcomes [17,31,32]. Based on this evidence, we consider the 48 h interval sufficient to prevent residual fatigue while preserving the intended benefits of familiarization.

**Figure 1:**
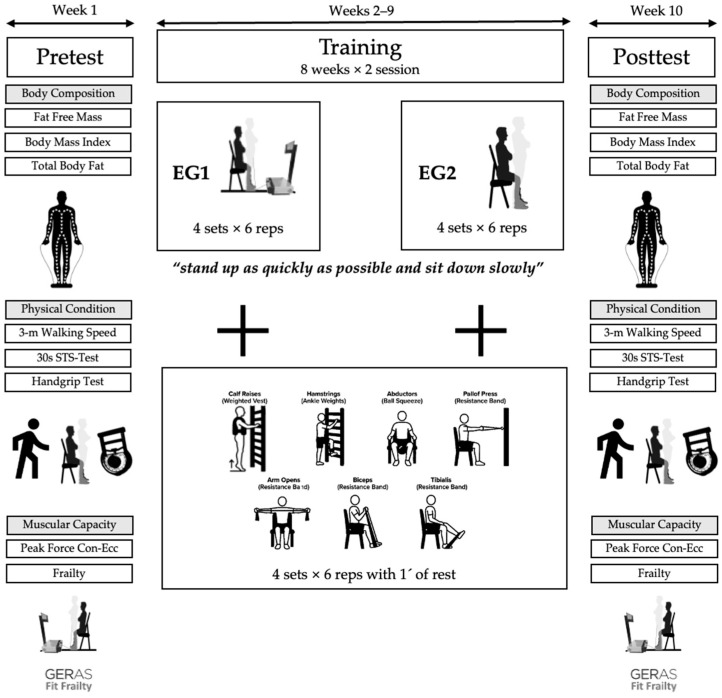
Strength training program.

The intervention lasted for two months, with a frequency of two sessions per week (Tuesdays and Thursdays), with sessions lasting approximately 45–60 min (Figure 1).

The training consisted of five lower-body exercises and three upper-body exercises. Participants performed four sets of six repetitions for each exercise, with a three-minute rest between sets. They were encouraged to perform the downward (eccentric) phase of each movement in a controlled manner. The total training volume (sets × repetitions × load) was equally distributed across the intervention groups.

All participants began the training with an initial load of 3 kg. When participants were able to complete the prescribed sets and repetitions while maintaining the required intensity and recovering adequately between sessions, the load was increased by 0.5, 1, 1.5, or 2 kg in the following session, based on individual performance. This progression protocol was applied equally in both intervention groups. Participants had to stand up as quickly as possible and sit down slowly. When each subject completed the sets and repetitions while maintaining the required intensity and adequately recovering in each session. This series-by-series progression strategy has been shown to be effective in promoting rapid strength gains in previous short-term studies [33,34,35]. Participants were instructed to inform their trainers if they experienced any discomfort during the training. Regarding perceived effort, the use of the Borg Rating of Perceived Exertion Scale was excluded, as many participants presented cognitive impairment or dementia, which could compromise the reliability of self-reported exercise intensity [36,37].

#### Partial Blinding of Assessors

In our study, strength assessment using dynamometry was conducted by a researcher who did not participate in the training sessions, acting as a blinded evaluator for both the pre- and post-intervention measurements. This approach minimizes the risk of bias in this key variable. Previous studies employing blinded evaluator designs have been considered methodologically robust in interventions with older adults [38,39]. The other functional tests (chair-stand, gait speed, etc.) were performed by other team members, including the trainers. Although these evaluators were not blinded to group allocation, all tests followed standardized and objective protocols (repetitions, times, distances), thereby reducing the likelihood of measurement bias.

### 2.3. Experimental Protocol

#### 2.3.1. Body Composition and Physical Activity Habits

A basic anthropometric assessment of weight and height was conducted, as well as the Body Mass Index (BMI). The anthropometric measurements followed the protocols of the International Society for the advancement of Kinanthropometry (ISAK) [40]. Height was measured with participants standing barefoot using a portable stadiometer (Seca GmbH & Co., KG, Hamburg, Germany) with an accuracy of 0.1 cm. Weight and body composition was assessed using bioelectrical impedance analysis (BIA) with participants standing, without shoes or socks, using a Tanita MC-780 multifrequency segmental body composition analyzer (Amsterdam, The Netherlands) with an accuracy of 0.1 kg.

The variables obtained from BIA included fat mass (FM), fat-free mass (FFM), total body water (TBW), visceral fat index, daily caloric intake (DCI), and basal metabolic rate (BMR). BMI was calculated by dividing body weight (kg) by height squared (m^2^). This BIA technology has been recently validated in adults with varying levels of physical activity [41]. Visceral fat was indirectly estimated and expressed using a specific rating scale ranging from 0 to 59 arbitrary units (AU), where values from 1 to 12 indicate a healthy level of visceral fat, and values from 13 to 59 indicate excess visceral fat [42].

Therefore, the Physical Activity Scale for the Elderly (PASE) was administered to collect data on physical activity, household tasks, and occupational activities performed over the previous seven days. This scale was specifically developed to assess physical activity levels in epidemiological studies involving individuals aged 65 years and older. In addition, PASE scores are commonly used as part of frailty assessment protocols, as reduced physical activity is a key component of the frailty phenotype [43]. In this study, the PASE was used to characterize baseline physical activity levels and to complement frailty assessment, providing contextual information about participants’ activity habits.

#### 2.3.2. Handgrip Test

Handgrip strength (HG) was measured using a Jamar hand dynamometer (Sammons Preston Inc., Bolingbrook, IL, USA). Participants were instructed to stand and hold the dynamometer with their arm parallel to their body, ensuring it was not pressed against their body. Both hands were tested during this assessment. The width of the handle was adjusted to fit each participant’s hand, ensuring that the middle phalanx rested comfortably on the inner handle. Participants were allowed one practice trial, followed by three test trials, with the best score used for analysis. HG was expressed in kilograms (kg). Testing was conducted on both arms. A manual muscle strength of 30 kg or greater for men and 20 kg or greater for women was considered normal; values below these thresholds were deemed inadequate [44,45].

#### 2.3.3. Chair Stand Test

The participant begins seated in the middle of the chair, with their back straight, feet flat on the floor, and arms crossed over their chest. At the “go” signal, the participant should stand up completely and return to the initial position as many times as possible within 30 s. It is recommended to demonstrate the exercise slowly first, allowing the participant to observe the correct execution, followed by a faster demonstration for better understanding. Before starting the test, the participant should perform the exercise one or two times to ensure correct execution [46].

#### 2.3.4. Walking 3 Meter Speed

The walking speed over three meters was evaluated by timing how long each participant took to cover the distance [47]. Participants started behind a marked line, and at the signal, the stopwatch was activated as they began to walk. The stopwatch was stopped when the participant crossed the finish line with their last foot, both of which were marked on the ground. Two attempts were conducted, and the time from the best attempt was recorded and converted to meters per second for analysis, with a rest period of three minutes between repetitions.

#### 2.3.5. Strength Measurement

Participants sat on a firm, upright chair (measuring 40 cm in height) with their arms crossed, and their hip, knee, and ankle joints were positioned at roughly 90 degrees. Following the countdown of 3, 2, 1, participants were instructed to initiate the concentric phase at maximum velocity upon command from this position. Concentric and eccentric peak force was measured using a sit-to-stand test with 5 repetitions on the FEMD (Myoquality M1, Myoquality Solutions, Granada, Spain [17,48]. The weight set to complete the set was 3 kg. The FEMD was set up in ‘Tonic Mode’, which featured an external load designed to mimic a free weight.

The linear displacement of each participant was measured while standing, using a FEMD rope attached to a harness secured to their waistcoat. The maximum force in kilograms for each repetition was captured during both the concentric and eccentric phases via the FEMD software Myosoft 2.8.4. The test was considered reliable if the knees and trunk were fully extended at the conclusion of the concentric phase [17].

Both muscular strength and power were determined using a FEMD (Myoquality M1, Myoquality Solutions, Granada, Spain). The peak concentric and eccentric force during the sit-to-stand movement was measured, using a reliable instrument for squat protocols, allowing for the reliable evaluation of these parameters [31]. The operation of the FEMD for obtaining these variables involves the performer applying forces to a rope wound around a roller, controlling and measuring both force and linear velocity [18]. A load cell detects the tension applied to the rope, and the resulting signal is converted from analog to digital with a resolution of 12 bits. Speed and displacement data are obtained using a 2500 ppr encoder coupled to the roller. The sensors collect various data at a frequency of 1 kHz [31].

### 2.4. Statistical Analysis

Statistical analyses were conducted using SPSS (Version 29.0; IBM Corp., Armonk, NY, USA). Descriptive statistics were reported as means and standard deviations (SD) for continuous variables and percentages for categorical variables. Pre-intervention differences among the three groups (EG1, EG2, CG) were examined using paired-sample t tests or analysis of variance (ANOVA). The Shapiro–Wilk test was applied to assess the normality of continuous dependent variables across groups.

A mixed-model ANOVA, which integrates between-subject and within-subject factors, was employed to evaluate interaction effects between moment (pre vs. post) and group (EG1, EG2, CG). This approach is widely recognized as standard practice for assessing intervention effects in exercise physiology [49]. Compared to the general linear model, mixed models offer superior handling of missing data in repeated-measures designs [50]. Accordingly, mixed-model ANOVA was used to estimate intervention effects based on significant moment × group interactions. Analyses adhered to the intent-to-treat principle.

**Figure 2:**
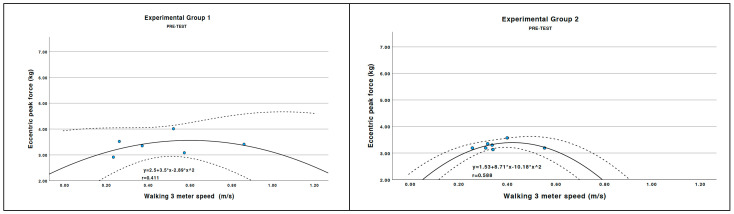

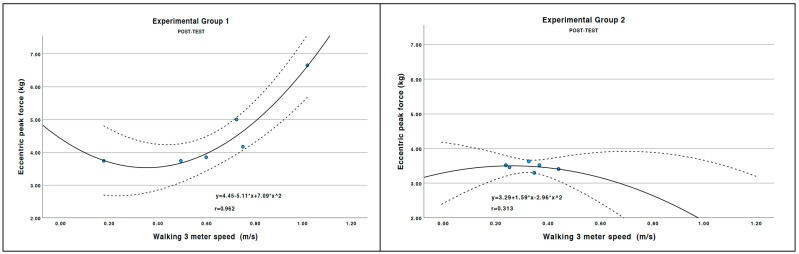
Correlations between peak eccentric force and walking speed in both strength intervention groups during the pre-test and post-test.

Graphical representations (Figure 2) illustrate the relationship between 3 m walking speed (m/s) and eccentric peak force (kg) at pre- and post-intervention across experimental groups. Statistical significance was set at *p* < 0.05 (two-tailed). Post hoc comparisons were conducted using the Bonferroni adjustment.

## 3. Results

### 3.1. Physical Characteristics of the Sample

The physical characteristics of the sample are presented in Table 1. The results for the three groups (EG1, CC, and EG2) before and after the intervention are detailed in Table 2, focusing on variables related to body composition, physical condition, and muscular strength.

The mixed-model ANOVA (2 × 3) showed no significant group × moment interactions for any of the variables related to body composition, physical condition, or muscular capacity (Table 2), indicating that the patterns of change between groups did not differ statistically. However, main effects of group and moment were observed for some variables; therefore, the within-group analyses are presented solely as complementary descriptive information and do not imply differences in the relative effectiveness of the interventions.

### 3.2. Body Composition

The mixed-model ANOVA revealed no significant group × moment interactions for any body composition variables (*p* > 0.05), indicating that the patterns of change did not differ statistically between groups. Within-group analyses showed that EG1 experienced a small but statistically significant increase in fat-free mass (+2.0 kg, *p* < 0.05) and BMI (+1.2 kg/m^2^, *p* < 0.05), while no significant changes were observed in total body fat or body water (*p* > 0.05). Conversely, EG2 exhibited a significant increase in total body fat (+1.2 kg, *p* < 0.05), with no other meaningful changes. The control group (CG) did not present significant variations in any body composition parameter (*p* > 0.05).

### 3.3. Functional Variables

The mixed-model ANOVA indicated no significant group × moment interactions for physical function (3 m walking speed *p* = 0.423; Sit-to-Stand 30 s *p* = 0.388; handgrip dominant *p* = 0.827; handgrip non-dominant *p* = 0.838). There were main effects of group for walking speed (*p* = 0.011) and Sit-to-Stand (*p* = 0.035), while moment effects were not significant (all *p* > 0.40). Within groups, EG1 improved walking speed (0.5 ± 0.2 to 0.6 ± 0.3 m/s; ≈+0.1 m/s; significant) and Sit-to-Stand performance (10.3 ± 2.5 to 15.2 ± 4.4 repetitions; +4.9; significant). Handgrip (dominant) decreased in EG1 (21.4 ± 8.3 to 20.2 ± 8.0 kg; −1.2 kg; significant), with no significant change in the non-dominant hand (20.6 ± 8.4 to 19.5 ± 7.3 kg). EG2 and CG did not show significant pre–post changes in walking speed or Sit-to-Stand; descriptively, CG walking speed declined (0.5 ± 0.1 to 0.4 ± 0.1 m/s).

### 3.4. Muscular Capacity

The mixed-model ANOVA revealed no significant group × moment interactions for frailty (*p* = 0.106), concentric peak force (*p* = 0.109), or eccentric peak force (*p* = 0.446). There was a main effect of moment for frailty (*p* = 0.001) and concentric peak force (*p* = 0.043), and main effects of group for concentric (*p* = 0.040) and eccentric peak force (*p* = 0.046). Complementary within-group descriptives showed that EG1 increased concentric peak force (4.8 ± 0.4 to 7.0 ± 1.3 kg; +2.2 kg; significant) and eccentric peak force (3.4 ± 0.4 to 4.5 ± 1.1 kg; +1.1 kg; significant). EG2 increased eccentric peak force (3.3 ± 0.1 to 3.5 ± 0.1 kg; +0.2 kg; significant), with a modest, non-significant rise in concentric force (4.5 ± 0.2 to 4.9 ± 0.6 kg). CG showed a small, non-significant decline in concentric peak force (4.8 ± 0.3 to 4.6 ± 0.6 kg) and a slight increase in eccentric force (3.4 ± 0.2 to 3.5 ± 0.2 kg). Frailty scores worsened descriptively in CG (26.6 ± 8.3 to 32.4 ± 5.6) and improved in EG1 (23.0 ± 6.3 to 20.3 ± 6.3) and EG2 (28.8 ± 3.3 to 27.6 ± 3.7), consistent with the moment main effect but not supported by a significant interaction.

Post hoc Bonferroni comparisons (Table 3) revealed no statistically significant differences between EG1 and CG in walking speed (*p* = 0.053), although EG2 performed significantly better than EG1 (mean difference = −0.31 m/s, *p* = 0.005). The comparison between EG2 and CG was not significant (*p* = 1.000). For the Sit-to-Stand test, EG1 performed significantly better than CG (mean difference = 7.13 repetitions, *p* = 0.017), whereas the difference between EG1 and EG2 did not reach statistical significance (*p* = 0.076). Regarding frailty, EG1 showed significantly lower frailty scores compared with CG (mean difference = −13.25, *p* < 0.001), and EG2 also performed significantly better than CG (mean difference = −8.83, *p* = 0.008), with no significant difference between EG1 and EG2 (*p* = 0.125). For neuromuscular outcomes, EG1 demonstrated significantly greater concentric peak force than both CG (mean difference = 1.60 kg, *p* = 0.030) and EG2 (mean difference = 1.40 kg, p = 0.016). In terms of eccentric peak force, EG2 showed significantly higher values than EG1 (mean difference = −0.95 kg, *p* = 0.026), whereas the difference between EG1 and CG did not reach statistical significance (*p* = 0.157).

### 3.5. Relationship Between Walking Speed and Peak Eccentric Force

Figure 2 illustrates the effects of the intervention on peak eccentric force and its relationship with walking speed in the strength training groups. In the post-test, EG1 demonstrated better application of eccentric force; higher walking speed corresponded to greater eccentric force application, while EG2 did not display stable behavior. EG1 utilized higher values of eccentric force during walking. Both experimental groups increased peak eccentric force. In the post-test, EG1 exhibited a stronger association between eccentric peak force and walking speed, suggesting a potential relationship between neuromuscular adaptations and functional performance; however, causal inferences cannot be established.

## 4. Discussion

The objective of this pilot study was to analyze the effects of two strength interventions focusing on the eccentric phase on peak strength (concentric and eccentric) in older adults residing in a nursing home, as well as to assess their impact on functional capacity and health-related indicators of frailty and independence.

The main findings highlight that EG1 achieved significant improvements in concentric and eccentric peak force, walking speed, and chair stand performance, while EG2 showed smaller gains. The mixed-model ANOVA did not reveal significant group × moment interactions for concentric or eccentric peak force. However, significant main effects of group and Bonferroni-adjusted post hoc comparisons supported between-group differences in concentric peak force. Therefore, differential intervention effects between groups cannot be statistically established. Although significant main effects of group and Bonferroni-adjusted post hoc comparisons were observed for concentric peak force, these findings should be interpreted with caution and do not indicate superiority of one intervention over another. These results are consistent with previous studies emphasizing the role of eccentric training in enhancing neuromuscular efficiency [20] and functional independence in older adults [24,25], particularly at the within-group level.

Frailty indicators decreased in EG1, although not reaching statistical significance, while Bonferroni comparisons confirmed that EG1 differed significantly from CG. This supports the idea that even modest changes in frailty scores may translate into meaningful health gains, as reported by Studenski et al. [2] and van Kan et al. [6], who identified gait speed and frailty indicators as predictors of hospitalization, falls, and loss of autonomy. Our results suggest that short-term, low-frequency resistance training using a functional electromechanical dynamometer (FEMD) may represent a promising strategy for addressing frailty-related outcomes, supporting independence, and improving quality of life, in line with previous evidence on functional resistance training [3,4]. However, these results derive from a very small sample size and therefore are not generalizable to broader populations.

The improvements in walking speed observed in this study, together with gains in eccentric and concentric force, underscore the potential of tailored strength training as a non-pharmacological intervention for maintaining health, independence, and well-being among frail older adults.

This agrees with findings from Cadore et al. [4] and Fragala et al. [51], emphasized that muscle power and walking speed are critical markers of functional capacity and predictors of adverse outcomes. In our study, Bonferroni tests confirmed significant differences between EG1 and EG2 in walking speed, suggesting a potential functional advantage of FEMD-based training for mobility-related outcomes.

These findings reinforce that functional resistance training may represent a low-cost and sustainable approach, as highlighted by Tou et al. [52] and Ramírez-Campillo et al. [53], demonstrated that high-speed resistance training improves functionality in frail and pre-frail older adults.

All participants performed the same eight exercises twice a week for 8 weeks. The only methodological difference between the two groups was the use of a weighted vest or a cord during the sit-to-stand exercise. The results indicate that both interventions achieved statistically significant differences in peak eccentric strength, with EG1 also showing improvements in concentric strength. This supports the idea that eccentric-focused interventions are particularly effective, consistent with Caserotti et al. [23] and Roig et al. [24].

The second objective was to determine whether strength improvements affected functional capacity in sedentary and older adults residing in a nursing home; in this case, the intervention with the FEMD resulted in significant improvements in the Chair Stand Test. These findings suggest that the intervention applied in EG1 was associated with improvements in selected functional outcomes, as strength gains were accompanied by improvements in walking speed and performance in the sit-to-stand task [46,54]—both of which are key indicators of mobility and independence in older adults.

In terms of body composition, no significant differences were observed between pre- and post-intervention measurements in the control group. In contrast, EG1 demonstrated increases in both body weight and BMI without a corresponding increase in fat mass, suggesting that these changes may be attributable to gains in fat-free mass. Although an increase in fat-free mass was observed in EG1, it did not reach statistical significance, possibly due to the small sample size or the short duration of the intervention.

Despite the potential impact on public health, there are few interventions aimed at mitigating the decline in functional capacity among institutionalized populations [38]. Physical activity, particularly strength training, is a crucial strategy for preventing or delaying frailty in older adults. Current literature highlights that high-speed resistance training is effective for improving functionality in older adults [4,5,52,53], as muscle power is a more sensitive predictor of functional capacity than isolated maximum strength. The results of this study support this exercise prescription, as EG1 demonstrated improvements in both peak concentric and eccentric force, while EG2 showed smaller improvements.

To our knowledge, this is the first intervention conducted with a FEMD in older adults residing in a nursing home. The use of this device appears to offer advantages in controlling load throughout the movement (particularly during the eccentric phase), which may explain the greater within-group gains in peak strength were observed in EG1 (46.5% concentric; 34% eccentric) compared with EG2, although these differences should be interpreted cautiously due to the absence of significant group × moment interactions (4.4% and 6.1%, respectively). The differences between the two interventions likely lie in biomechanical aspects and execution, as using the FEMD requires participants to remain under tension longer, especially in the eccentric phase, potentially enhancing body awareness and control throughout the movement. This may facilitate optimal progressive overload and an increasing workload, generating greater mechanical tension through Maximum Voluntary Contraction (MVC).

The improvement in strength gains induced by training is also associated with enhancements in muscular function. EG1 showed significant improvements in the 30 s sit-to-stand test and walking speed, while EG2 did not exhibit significant differences in these tests. In this context, several studies [54,55,56] advocate for the use of rapid execution training to maximize the effects of exercise therapies in older individuals, including the oldest old [57]. Reid & Fielding [58] explain that power—the ability to generate force quickly—is crucial for performing daily movements and responding to situations requiring rapid reactions, such as preventing falls. They highlight how this type of training can induce neural changes in muscle recruitment that transfer to both isometric and dynamic exercises [56,59]. Lavin et al. [60] suggest that neuromuscular aging can be inhibited by improving motor unit integrity, and corticospinal excitability, while Maki & McIlroy [61] emphasized the importance of rapid torque generation for balance recovery.

Regarding training with a weighted vest, the study by Mierzwick [62] reported improvements in certain strength variables but no significant differences in the five-time sit-to-stand test. One explanation is that the load from the vest applies to the trunk and pelvis rather than directly to the lower limb. Pagan et al. [35] also noted that vest training provides overload benefits when worn during daily activities or aerobic resistance, but not for maximum strength improvement.

In terms of upper body strength, no improvements were observed in either EG1 or EG2, as seen in other studies [63,64]. Furthermore, the EG1 group exhibited a decrease in hand grip strength in the dominant hand. This decrease could be attributed to the greater emphasis on overload for other body parts, with insufficient direct stimulation of the forearm and hand musculature due to the use of bands and the sets and repetitions outlined in this study. This highlights the importance of including specific exercises for upper limb strength if improvement in this area is desired, as Valdés-Badilla et al. [63] and Chen et al. [64] also reported limited gains in grip strength when upper limb training was not specifically targeted.

Concerning frailty, there was a trend toward improvement in EG1 (decreasing from 23.00 to 20.33, *t* = 2.090, *p* = 0.091), although it did not reach statistical significance. This suggests that longer interventions or larger samples may be required to achieve statistical significance. Nevertheless, even small individual improvements in frailty scores can represent meaningful gains in daily life, as gait speed and frailty indices are strongly associated with autonomy and hospitalization risk [2,6].

The control group showed no significant changes in any evaluated parameters, including body composition, physical condition, and muscular capacity. This supports the idea that strength training is the determining factor for the improvements observed in EG1, as the absence of training stimulus resulted in no modifications in the evaluated variables. Moreover, the strength gains in EG2 did not reach a minimal effective load necessary to translate into improvements in functional capacities that influence the quality of life of older adults.

Walking speed has been recognized in scientific literature as a key marker of functional capacity and overall health, particularly in older adult populations. Studies have reported that increases in muscle power contribute to improved force generation speed, associated with greater walking speed, better quality of life, and reduced frailty (or lower risk of hospitalization, falls, and loss of autonomy) [2,6,51].

In our study, the correlation between eccentric peak force and walking speed was confirmed, reinforcing the idea [26,51] that eccentric adaptations are critical for mobility improvements.

The current results, demonstrating that even with a frequency of two sessions per week, significant improvements in muscle strength can be achieved, which is essential for maintaining autonomy in this population. During the eccentric phase, the muscle lengthens while maintaining activated actin and myosin bridges under tension. This process not only controls impact upon ground contact but also stores elastic energy in muscle and tendon structures. This energy is then released in the concentric phase, where the muscle shortens to propel movement. This interaction is key to movement efficiency and economy. The improved application of force during movement appears to stem from enhanced Stretch-Shortening Cycle capabilities in those who trained with the dynamometer compared to those who underwent weighted vest training. One possible explanation for these gains may be that participants using the dynamometer experienced more time under tension than those training with the vest [17]. Therefore, gains in the eccentric phase, along with those in the concentric phase, may have led to improvements in functional tests (greater capacity to transfer forces) [26]. Inability to fully activate a muscle occurs when there is inadequate neural input for a muscle or when excitation-contraction coupling is insufficient to ensure that all muscle motor units are active and firing at the appropriate maximum frequency [59]. Conversely, the EG2 group demonstrated less stable behavior, indicating that adaptations in the stretch-shortening cycle (and thus in eccentric phase effectiveness) were neither consistent nor significant. In conclusion, it can be suggested that improvements in eccentric strength optimize the transition to the concentric phase, allowing for more effective utilization of stored elastic energy. However, improvements in the concentric phase are also necessary for enhancing functional capacity in dependent and institutionalized older adults.

This study has several limitations that should be considered when interpreting the results. First, the small sample size and the presence of various comorbidities—particularly those related to cognitive impairment—limited the ability to maintain strict control over training load and adherence. Additionally, the absence of dietary monitoring or nutritional intervention may have influenced the outcomes related to body composition. Finally, the lack of post-intervention follow-up makes it difficult to assess the long-term sustainability of the improvements achieved.

Another possible limitation is the “attention effect” in the control group, since participants may benefit from social interaction or minimal activity even without structured training. Future studies should consider allowing control groups to engage in light activities (e.g., social gatherings, stretching) to minimize this bias. Moreover, the absence of blinding for evaluators represents another methodological limitation that should be addressed in subsequent trials.

## 5. Conclusions

This pilot study suggests that eccentric-focused strength training performed with a functional electromechanical dynamometer is associated with neuromuscular improvements, particularly in concentric peak force, in frail nursing home residents. However, no significant group × moment interactions were observed, indicating that differential training effects between interventions cannot be established. Post hoc between-group comparisons therefore reflect cross-sectional differences rather than training-induced superiority. Functional improvements should be interpreted cautiously.

FEMD-based training may represent a promising functional strength training strategy for institutionalized older adults. Future randomized controlled trials with larger samples and longer follow-up are required to confirm these findings.

## Figures and Tables

**Table 1:** Descriptive analysis of the body composition in older adults organized by groups.

	All (n = 19)	CG (n = 7)	EG1 (n = 6)	EG2 (n = 6)
	Mean ± SD	Mean ± SD	Mean ± SD	Mean ± SD
Age (years)	86.7 ± 5.8	86.2 ± 7.8	86.2 ± 5.9	87.4 ± 4.3
Weight (kg)	64.1 ± 10.6	68.3 ± 15.4	62.8 ± 9.4	61.6 ± 6.4
Height (cm)	153.6 ± 6.8	154.9 ± 7.4	152.0 ± 6.3	153.9 ± 7.5
Waist Hip Ratio	0.9 ± 0.1	0.96 ± 0.0	0.9 ± 0.1	0.9 ± 0.0
Sex, female (%)	63.1	71.4	66.70	50.0

Legend: CG = Control Group; EG1 = Experimental group with dynamometer; EG2 = Experimental group with weighted vest.

**Table 2:** Performance Changes in Training and Control Groups Following 8-Week Intervention in Dwelling Older Adults (Mixed ANOVA 2 × 3).

	Control Group (CG)		Experimental Group 1 (EG1)		Experimental Group 2 (EG2)		ANOVA MIXED (2 × 3)		
	Pre Mean ± SD	Post Mean ± SD	Pre Mean ± SD	Post Mean ± SD	Pre Mean ± SD	Post Mean ± SD	Group Sig	Moment Sig	Group × Moment Sig
**Body Composition**									
Fat-Free Mass (kg)	50.0 ± 4.4	47.6 ± 6.4	40.9 ± 5.8	42.9 ± 6.0 *	38.9 ± 7.5	39.7 ± 8.6	0.192	0.443	0.843
BMI (kg/m^2^)	29.2 ± 8.3	33.2 ± 13.1	27.1 ± 2.8	28.3 ± 3.6 *	26.1 ± 2.3	26.6 ± 2.8	0.569	0.435	0.759
Total Body Fat (kg)	22.6 ± 14.6	25.9 ± 17.9	22.5 ± 2.7	20.6 ± 7.5	19.7 ± 6.1	20.9 ± 5.5 *	0.468	0.205	0.512
Body Water (kg)	33.4 ± 4.6	35.5 ± 5.5	30.0 ± 4.0	30.7 ± 4.5	28.2 ± 5.2	29.1 ± 4.9	0.116	0.387	0.780
Visceral Fat Indicator	16.2 ± 4.7	18.3 ± 3.1	13.0 ± 4.7	12.8 ± 4.3	12.7 ± 2.1	13.8 ± 2.6	0.790	0.998	0.917
DCI/BMR (kcal)	1421.6 ± 165.1	1512.3 ± 167.7	1282.5 ± 154.6	1295.4 ± 172.8 *	1206.7 ± 172.4	1257.5 ± 178.0	0.175	0.548	0.895
**Physical Condition**									
3 m Walking speed (m/s)	0.5 ± 0.1	0.4 ± 0.1	0.5 ± 0.2	0.6 ± 0.3 *	0.4 ± 0.1	0.4 ± 0.1	0.011	0.410	0.423
Sit and Stand 30 s (n)	9.2 ± 4.8	6.7 ± 1.7	10.3 ± 2.5	15.2 ± 4.4 *	8.7 ± 2.4	10.0 ± 3.7	0.035	0.470	0.388
Handgrip (Dominant, kg)	20.5 ± 5.7	14.0 ± 7.3	21.4 ± 8.3	20.2 ± 8.0 *	17.3 ± 5.2	17.1 ± 3.8	0.114	0.432	0.827
Handgrip (Non-Dominant, kg)	20.7 ± 5.7	11.7 ± 4.7	20.6 ± 8.4	19.5 ± 7.3	15.6 ± 4.0	15.3 ± 3.4	0.100	0.409	0.838
**Muscular**									
Frailty Indicator	26.6 ± 8.3	32.4 ± 5.6	23.0 ± 6.3	20.3 ± 6.3	28.8 ± 3.3	27.6 ± 3.7	0.187	0.001	0.106
Concentric Peak Force (kg)	4.8 ± 0.3	4.6 ± 0.6	4.8 ± 0.4	7.0 ± 1.3 *	4.5 ± 0.2	4.9 ± 0.6	0.040	0.043	0.109
Eccentric Peak Force (kg)	3.4 ± 0.2	3.5 ± 0.2	3.4 ± 0.4	4.5 ± 1.1 *	3.3 ± 0.1	3.5 ± 0.1 *	0.046	0.142	0.446

Legend: BMI = Body mass index; CG = Control Group; DCI/BMR = Daily Caloric Intake and Basal Metabolic Rate; EG1 = Experimental group with dynamometer; EG2 = Experimental group with weighted vest; * = sig > 0.05.

**Table 3:** Post-Anova Intervention-up Test Using Bonferroni.

Multiple Comparison								
Dependent Variable		Groups	Groups	Mean Difference	Std Error	Sig.	95% Confidence Interval	
							Lower Limit	Upper Limit
3 m Walking speed (m/s)	Bonferroni	EG1	CG	0.2714	0.10190	0.053	−0.0031	0.5458
			EG2	0.3089	0.08086	0.005	0.0911	0.5267
		CG	EG1	−0.2714	0.10190	0.053	−0.5458	0.0031
			EG2	0.0375	0.09999	1.000	−0.2318	0.3069
		EG2	EG1	−0.3089	0.08086	0.005	−0.5267	−0.0911
			CG	−0.0375	0.09999	1.000	−0.3069	0.2318
Sit and Stand 30 s (n)	Bonferroni	EG1	CG	7.1250	2.20605	0.017	1.1825	13.0675
			EG2	4.3472	1.75048	0.076	−0.3681	9.0626
		CG	EG1	−7.1250	2.20605	0.017	−13.0675	−1.1825
			EG2	−2.7778	2.16481	0.657	−8.6092	3.0537
		EG2	EG1	−4.3472	1.75048	0.076	−9.0626	0.3681
			CG	2.7778	2.16481	0.657	−3.0537	8.6092
			CG	0.7778	3.81323	1.000	−9.4941	11.0496
Frailty Indicator	Bonferroni	EG1	CG	−13.2500	2.49727	<0.001	−19.9770	−6.5230
			EG2	−4.4167	1.98156	0.125	−9.7545	0.9211
		CG	EG1	13.2500	2.49727	<0.001	6.5230	19.9770
			EG2	8.8333	2.45059	0.008	2.2321	15.4346
		EG2	EG1	4.4167	1.98156	0.125	−0.9211	9.7545
			CG	−8.8333	2.45059	0.008	−15.4346	−2.2321
Concentric Peak Force (kg)	Bonferroni	EG1	CG	1.5963 *	0.54189	0.030	0.1365	3.0560
			EG2	1.3999 *	0.42998	0.016	0.2416	2.5581
		CG	EG1	−1.5963 *	0.54189	0.030	−3.0560	−0.1365
			EG2	−0.1964	0.53176	1.000	−1.6288	1.2360
		EG2	EG1	−1.3999 *	0.42998	0.016	−2.5581	−0.2416
			CG	0.1964	0.53176	1.000	−1.2360	1.6288
Eccentric Peak Force (kg)	Bonferroni	EG1	CG	0.8313	0.39435	0.157	−0.2310	1.8935
			EG2	0.9460 *	0.31291	0.026	0.1031	1.7889
		CG	EG1	−0.8313	0.39435	0.157	−1.8935	0.2310
			EG2	0.1147	0.38698	1.000	−0.9277	1.1571
		EG2	EG1	−0.9460 *	0.31291	0.026	−1.7889	−0.1031
			CG	−0.1147	0.38698	1.000	−1.1571	0.9277

Legend: CG = Control Group; EG1 = Experimental group with dynamometer; EG2 = Experimental group with weighted vest; * = sig < 0.05.

## Data Availability

The data supporting the findings of this study are not publicly available due to ethical and privacy restrictions related to the protection of participants’ personal data. The data may be made available from the corresponding author upon reasonable request and with approval from the relevant ethics committee.
